# Supplementary material for: Yamogenin-Induced Cell Cycle Arrest, Oxidative Stress, and Apoptosis in Human Ovarian Cancer Cell Line
Source: Molecules. 2022 Nov 24;27(23):8181. doi: 10.3390/molecules27238181 (PMC9740764; doi:10.3390/molecules27238181)
Supplement: Supplementary file 1 [file molecules-27-08181-s001.zip › molecules-2026621-supplementary.pdf]

Table S1. The tested genes from the TaqMan Array Human Apoptosis Fast 96 well plates.

Gene symbol											
18S (ctrl)	GAPDH (ctrl)	HPRT1 (ctrl)	GUSB (ctrl)	**BIRC2**	**APAF1**	**BAD**	**BAK1**	**BAX**	**BBC3**	**BCAP31**	**BCL10**
**BCL2**	**BCL2A1**	**BCL2L1**	**BCL2L10**	**BCL2L11**	**BCL2L13**	**BCL2L14**	**BCL2L2**	**BCL3**	**BID**	**BIK**	**NAIP**
**BIRC3**	**XIAP**	**BIRC5,EPR1**	**BIRC6**	**BIRC7**	**BIRC8**	**BNIP3**	**BNIP3L**	**BOK**	**NOD2**	**NOD1**	**CARD6**
**CARD9**	**CASP1**	**CASP10**	**CASP14**	**CASP2**	**CASP3**	**CASP4**	**CASP5**	**CASP6**	**CASP7**	**CASP8**	**CASP8AP2**
**CASP9**	**CFLAR**	**CHUK**	**CRADD**	**DAPK1**	**DEDD**	**DEDD2**	**DIABLO**	**IFT57**	**FADD**	**FAS**	**FASLG**
**HIP1**	**HRK**	**HTRA2**	**CARD18**	**IKBKB**	**IKBKE**	**IKBKG**	**LRDD**	**LTA**	**LTB**	**MCL1**	**NLRP1**
**NFKB1**	**NFKB2**	**NFKBIA**	**NFKBIB**	**NFKBIE**	**NFKBIZ**	**PEA15**	**PMAIP1**	**PYCARD**	**REL**	**RELA**	**RELB**
**RIPK1**	**RIPK2**	**TBK1**	**TNF**	**TNFRSF10A**	**TNFRSF10B**	**TNFRSF1A**	**TNFRSF1B**	**TNFRSF21**	**TNFRSF25**	**TNFSF10**	**TRADD**
